# Temporal disease trajectories condensed from population-wide registry data covering 6.2 million patients

**DOI:** 10.1038/ncomms5022

**Published:** 2014-06-24

**Authors:** Anders Boeck Jensen, Pope L. Moseley, Tudor I. Oprea, Sabrina Gade Ellesøe, Robert Eriksson, Henriette Schmock, Peter Bjødstrup Jensen, Lars Juhl Jensen, Søren Brunak

**Affiliations:** 1 Center for Biological Sequence Analysis, Department of Systems Biology, Technical University of Denmark, Kemitorvet, Building 208, DK-2800 Kgs. Lyngby, Denmark; 2 NNF Center for Protein Research, University of Copenhagen, Blegdamsvej 3B, DK-2200 Copenhagen, Denmark; 3 Department of Internal Medicine, University of New Mexico, MSC10 5550, 1 University of New Mexico, Albuquerque, New Mexico 87131, USA; 4 Department of Rheumatology and Inflammation Research, University of Gothenburg, Box 480, SE-40530 Gothenburg, Sweden; 5 Institute of Biological Psychiatry, Mental Health Center Sct. Hans, Copenhagen University Hospital, Boserupvej 2, DK-4000 Roskilde, Denmark

## Abstract

A key prerequisite for precision medicine is the estimation of disease progression from the current patient state. Disease correlations and temporal disease progression (trajectories) have mainly been analysed with focus on a small number of diseases or using large-scale approaches without time consideration, exceeding a few years. So far, no large-scale studies have focused on defining a comprehensive set of disease trajectories. Here we present a discovery-driven analysis of temporal disease progression patterns using data from an electronic health registry covering the whole population of Denmark. We use the entire spectrum of diseases and convert 14.9 years of registry data on 6.2 million patients into 1,171 significant trajectories. We group these into patterns centred on a small number of key diagnoses such as chronic obstructive pulmonary disease (COPD) and gout, which are central to disease progression and hence important to diagnose early to mitigate the risk of adverse outcomes. We suggest such trajectory analyses may be useful for predicting and preventing future diseases of individual patients.

## Abstract (web summary)

Population-wide analyses of healthcare data are challenging and thus often involve only few diseases or comparatively short time scales. Here the authors use healthcare records of all hospital encounters in Denmark over 15 years to describe how disease diagnoses progress over time.

## Graphical abstract

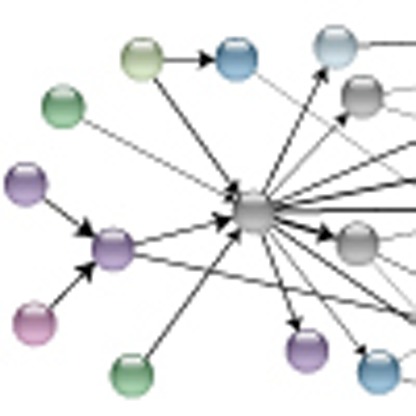

Population-wide analyses of disease correlations, co-morbidities and disease progression have so far mainly been carried out in a hypothesis-driven manner with focus on a few diseases^1^,^2^,^3^,^4^,^5^,^6^ or with focus on co-morbidities to index diseases^7^. Although useful in establishing near-term complications of the disease of interest, these studies are, by design, constrained to their closely limited and largely, already established complications. Recently, large-volume health record analysis was used to uncover associations between patterns of complex disease and Mendelian loci, demonstrating the validity of this modelling strategy^8^. Unfortunately, the nature of the registry data prevented the investigators from analysing the data using the key factor of time. This study focuses on frequently observed temporal patterns over the spectrum of pathologies of an entire country. Earlier data-driven studies have used a network approach to analyse data covering 3 years of Medicare claims, mainly Americans who are 65 years or older, which biased the analysis to geriatric diagnoses^9^,^10^.

The data foundation for the analysis is the Danish National Patient Registry (NPR), which covers all hospital encounters (inpatient admissions, outpatient visits and emergency room visits) of the entire Danish population for a 14.9-year period, from 1996 to 2010. Mandatory reporting from all Danish hospitals to the NPR is likely to severely limit the influence of population bias. This data set covers 6.2 million patients with a total of 65 million total clinical encounters, comprising 16 million hospital inpatient events (24.5% of total), 35 million outpatient clinic events (53.6% of total) and 14 million emergency department events (21.9% of total). Together, these encounters yielded 101 million unique assignments of diagnoses coded in the International Classification of Diseases (ICD-10) terminology.

Here we present the comprehensive set of time dependent, sequential diagnostic correlations that we have condensed from the entire population of Denmark. This set of sequential disease associations, which we define as disease trajectories, uncovers time–critical disease associations. They can also form the basis for understanding mathematical properties of co-morbidity networks^9^,^10^. Both types of studies highlight the potential of using patient registry data for exploring co-morbidities and the temporal and non-temporal patterns they display. However, the data analyses presented here may be more useful, as they exhibit trajectories that are thus amenable to interruption at various stages and they point out those stages.

The long time span and the large size of the data set allowed us to analyse disease progression in the form of diagnosis trajectories. From the data, we found 1,171 trajectories to have strong temporal directionality, statistical significance and thereby yield a global picture of the most populated, directional co-morbidities observed in the clinic. Among these are five groups of related trajectories, which allowed us to identify key diagnoses that can lead to severe outcomes and can thus be used to define groups of patients to include in comparative effectiveness research studies. Our analyses also showed the importance of stratifying a cohort into inpatient admissions, outpatient visits and emergency department visits.

## Results

### Importance of stratification for type of hospital encounter

**Figure 1:**
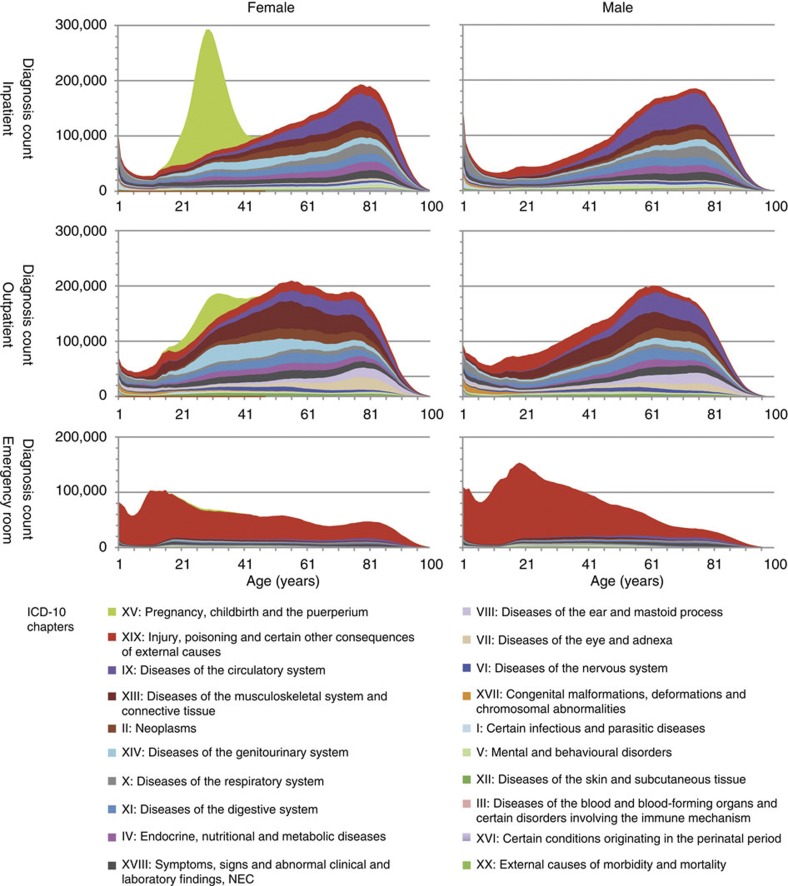
ICD-10 diagnoses from the National Danish Patient Registry covering the entire Danish population in the period 1996–2010. The data panels show females (left), males (right), inpatients (top), outpatients (middle) and emergency room patients (bottom). The colour-coding corresponds to ICD-10 chapter structure. The chapters are ordered so that the chapters with largest variance in diagnosis count is on top, starting with chapter XV ‘Pregnancy, childbirth and the puerperium’ and XIX ‘Injury, poisoning and certain other consequences of external causes’, 20 chapters in all.

Disease occurrences correlate strongly with age and gender, and thus it is an obvious necessity to correct for these underlying baseline biases. Figure 1 shows distributions of diseases across all 21 ICD-10 chapters in NPR stratified by age, gender and encounter type. Gender-specific differences in trends are clearly observable. Many diagnoses occur predominantly or exclusively in the inpatient, outpatient, or emergency department sites. Thus, a previously unrecognized and fundamentally important stratification is also by site of encounter. The fact that the NPR data set contains significantly more outpatient encounters (53.6% of total encounters) compared with inpatient and emergency department encounters (24.5 and 21.9% of total encounters, respectively) further supports the importance of this stratification strategy. Figure 1 summarizes the effect of site of encounter on disease trajectories. For males, injury codes stand out among young men (red), but Fig. 1 clearly shows that these diagnoses segregate much more strongly by encounter type than by gender and age. This example stands out as one of the strongest correlations between diagnoses and encounter type, but our analysis demonstrates that this trend holds true for most ICD-10 chapters. These data show that it is just as important to stratify diagnoses by the type of hospital encounter, as by age and gender. An important consideration in the subsequent analyses was therefore to make use of this aspect to stratify diagnosis assignments into more precisely matched groups. In this way, we enable both the discovery of statistically significant correlations that would have been otherwise masked and the removal of statistically significant correlations that are trivially explained by encounter types.

### Temporal co-morbidity analysis as basis for trajectories

To identify statistically significant, temporal correlations among pairs of diagnoses, we performed a cohort study where exposed patients who had a specific pair of diagnoses were matched with comparison patients with same age, gender and type of hospital encounter. We performed a pre-filtering step where initial *P*-values were estimated initially using a binomial test, and then confirmed using the full comparison group matching (see Methods).

From the full data set, we identified 1,194,343 pairs (D1→D2) of diagnoses where D2 occurs within a 5-year time frame of D1. From them, we excluded in total 370,737 codes related to pregnancy (chapters XV and XVI of ICD-10), general symptoms and signs not linked to a disease (chapter XVIII), external causes (chapters XIX and XX) and administration (chapter XXI). Among the 823,606 tested pairs, we identified 62,821 that were observed in at least 10 encounters, had relative risk (RR)>1 and were significant in the pre-filtering step with *P*<1.21 × 10^−9^ (binomial tests, Bonferroni corrected for multiple testing). This number increases by ~10,000 if patients are stratified only by age and gender, or by type of hospital encounter only, and by an additional ~8,000 when stratified by neither (see Table 1). Thus, stratification by type of hospital encounter is complementary to stratification by age and gender, and is equally important.

In addition to identifying pairs with increased risk, we tested for significant directionality: of the pairs D1→D2 with significant RR>1, we identified those where significantly higher number of patients had D1 occurring before D2 compared with the opposite direction or in the same admission. In all, 4,014 pairs were found to have a significant direction. All pairs were validated using the sampling model with a Bonferroni-corrected *P*-value of <1.21 × 10^−8^. Supplementary Data 1 lists all the pairs and their corresponding RR and *P*-values. The 4,014 directional pairs were then combined into longer trajectories consisting of three or more diagnoses. We identified a set of 5,784 trajectories with three diagnoses that cover between 1 and 16,197 patients in the last step. These were extended to 1,171 trajectories of four diagnoses covered by at least 20 patients in the last step, 1,077 (92%) of which have>99% bootstrap support. We further validated the trajectories by comparing them to a set of co-morbid pairs identified in a cohort of almost 600,000 individuals from the greater Stockholm area^11^. The trajectories capture 48% of the pairs, which is fourfold more than expected by chance (binomial test *P*=2.2 × 10^−16^). The entire set of 1,171 recurrent trajectories of four diagnoses each is shown in Supplementary Data 2.

### Clustering trajectories reveals disease development patterns

To produce a more comprehensive overview, we further clustered the trajectories based on which diagnoses they shared. As a similarity measure between diagnosis pairs, we used the Jaccard Index. The clustering identified 15 clusters; the 5 largest clusters covered 46, 25, 12, 9 and 8 diagnoses each, respectively. The five largest clusters were enriched for diseases of the prostate, chronic obstructive pulmonary disease (COPD), cerebrovascular disease, cardiovascular disease and diabetes mellitus, and for space limitations we focus on these below.

**Figure 2:**
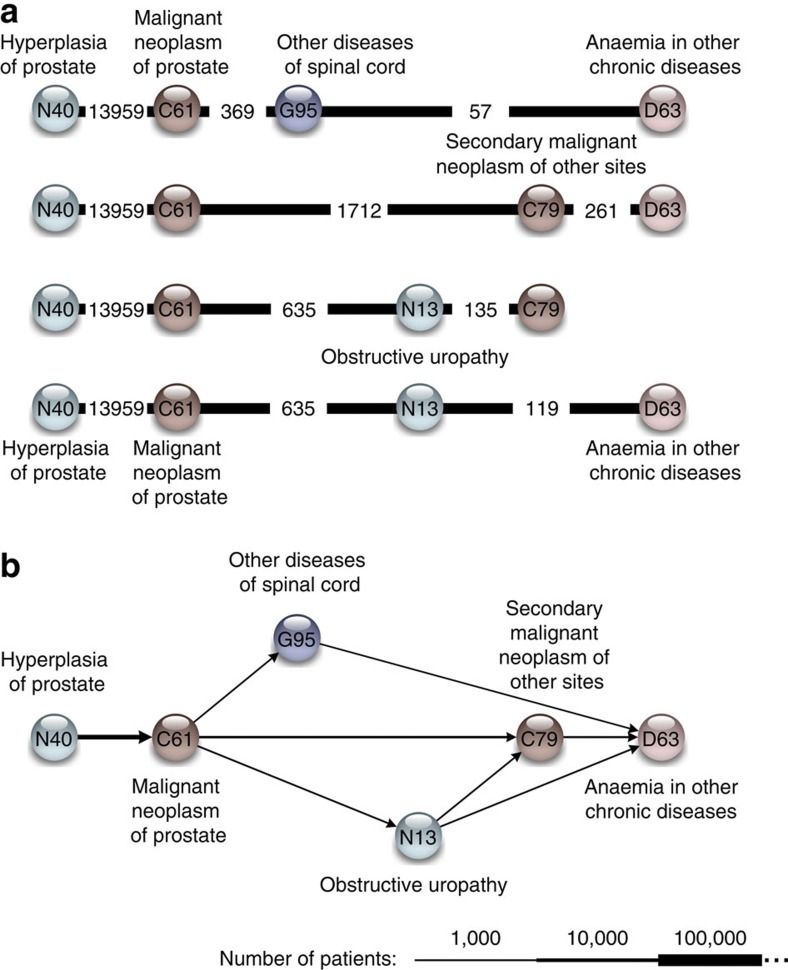
Disease trajectories and trajectory-cluster for prostate cancer. The figure illustrates the transition from trajectories to a trajectory cluster. Each circle represents a diagnosis and is labelled with the corresponding ICD-10 code. The colours represent different ICD-10 chapters. The temporal diagnosis progression goes from left to right. (**a**) All trajectories that contribute to the prostate-cancer cluster. The number of patients, who follow the trajectory until a given diagnosis, is given in the edges. (**b**) The prostate cancer trajectory cluster that represents all the trajectories. The width of the edges corresponds to the number of patients with the directed diagnosis pair from the full population. The cluster describes a normal progression from having hyperplesia of prostate diagnosed to having prostate cancer, cancer metastasis and anaemia.

The prostate disease cluster is the simplest, progressing from prostate hypertrophy (ICD-10 code: N40) through prostate cancer (C61) and obstructive uropathy (N13) to metastatic cancer (C79) and cancer-associated anaemia (D63) (Fig. 2). Except for the expected prostate-specific complications, this nearly linear trajectory cluster is representative of general cancer progression to metastasis and anaemia.

**Figure 3:**
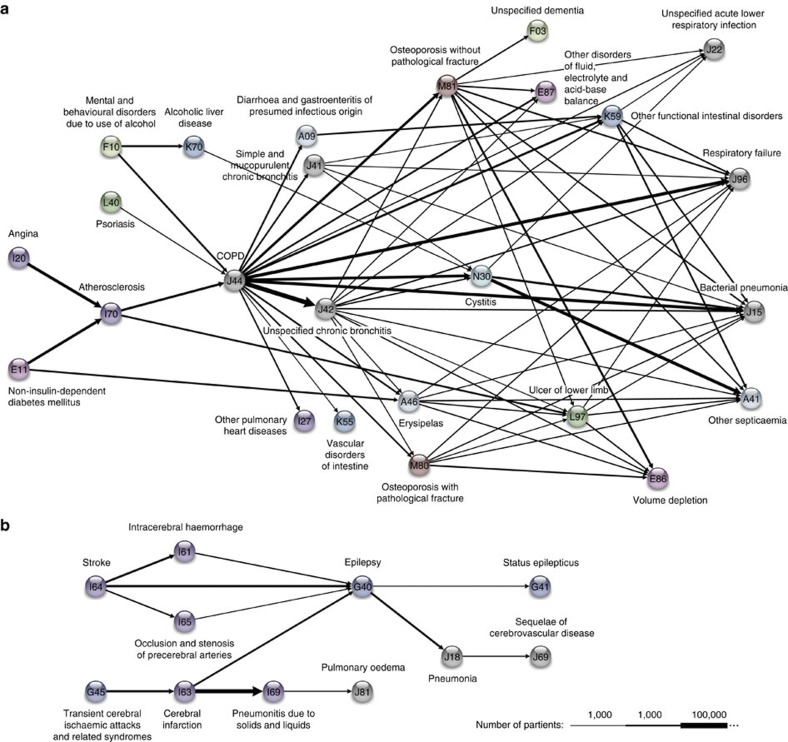
COPD and cerebrovascular disease trajectory clusters. (**a**) The COPD cluster showing five preceding diagnoses leading to COPD and some of the possible outcomes. (**b**) Cerebrovascular cluster with epilepsy as key diagnosis.

The COPD cluster has a characteristic structure, where a variety of diagnoses, including cardiovascular, skin, endocrine and behavioural disorders, converge on COPD (J44) and proceed to respiratory failure (J96), pneumonia (J15), septicaemia (A41) and other diagnoses (Fig. 3a). We tested whether COPD was a central diagnosis in the cluster by calculating the RR of COPD occurring between all diagnoses preceding and succeeding COPD, and found that COPD indeed had a large RR of 5.1 (sampling method *P*<10^−5^).

There is wide acceptance that cardiovascular disease-related morbidities are worsened in patients with COPD^2^,^12^,^13^,^14^. This association, however, generally considers the impact of cardiovascular events on pre-existing COPD or the co-existence of the two diagnoses. In contrast, our analysis demonstrates that a subsequent diagnosis of COPD has a profound impact on a number of cardiovascular diagnoses, whether angina pectoris (I20) or atherosclerosis (I70). In fact, all trajectories starting with atherosclerosis are followed by a subsequent COPD diagnosis, which supports the temporal pattern of diagnosis as well as pathophysiologic link. Once the diagnosis of COPD occurs, the disease trajectories tell a story of rapid progression (typically 1.8–2.5 years) to a variety of subsequent diagnoses. However, the most common outcome after COPD is death. Using a Kaplan–Meyer estimate, we found that 49.7% of patients following a trajectory containing COPD die within 5 years compared with 21.3% in a sex-, age- and encounter-matched comparison group. Over the full data period (14.9 years), 86.9% of these patients die while in the comparison group 36.2% of patients die. The high-mortality rate is confirmed in another study^15^: a 50% mortality at 3.6 years and 75% at 7.7 years from initial hospitalization.

**Figure 4:**
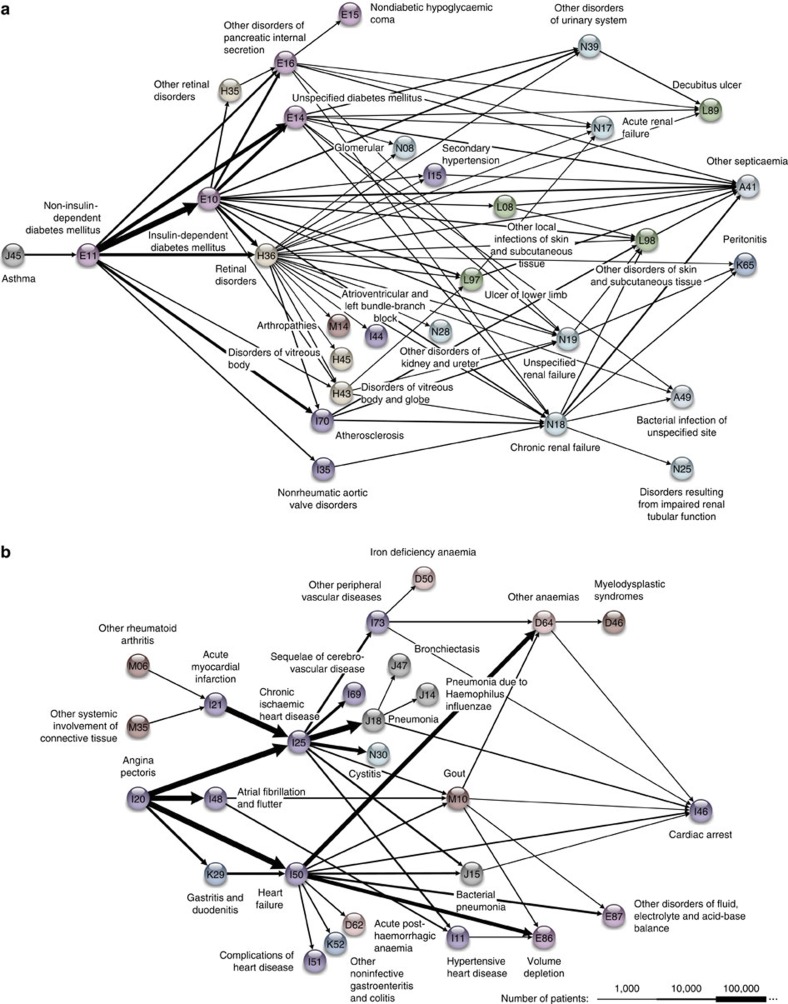
Diabetes and cardiovascular disease trajectory clusters. (**a**) Diabetes cluster showing progression from non-insulin-dependent to insulin-dependent diabetes. Retinal disorders are key diagnoses marking progression to worse conditions. (**b**) Cardiovascular cluster. A key finding is that gout is a central diagnosis in the cardiovascular cluster, supporting evidence that gout is important to progression of cardiovascular diseases in a keystone manner.

Similar to the COPD cluster, the cerebrovascular and diabetes clusters are characterized by convergence on key diagnoses, namely epilepsy (G40) and retinal disease (H36), respectively (Figs 3b and 4a). Epilepsy (with an RR of 6.6 for cerebrovascular disease, sampling method *P*<10^−5^) is likely to be a marker of significant cerebrovascular compromise^1^ reflecting the severity of the underlying disease. Similarly, retinopathy (RR=20.1, sampling method *P*<10^−5^ for diabetes) is a marker of the degree of system-wide diabetic vasculopathy^16^; population studies suggest that diabetic retinopathy is present in more than half of all diabetics^17^.

Gout (M10), similar to COPD and retinal disease, is a key diagnosis within the cardiovascular cluster (RR=6.8, sampling method *P*<10^−5^) and serves as central disease in a diabetes-independent cardiovascular diseases cluster (Fig. 4b). Associations between gout and cardiovascular disease have long been hypothesized^18^, and allopurinol has recently been suggested for management of cardiovascular disease^19^. In contrast, the recent CHARGE study failed to show a link between serum uric acid and cardiovascular risk^20^. Our population-wide trajectory data support the epidemiologic relationship between gout and cardiovascular diseases.

### Trajectories facilitate comparative effective research

In addition to providing an important analysis of temporal disease associations across an entire population, the trajectories and their associated networks offer a new paradigm to improve trial design for studies of comparative effectiveness. For example, the four diagnosis trajectories beginning with angina (I20) as first diagnosis and ending with cardiac arrest (I46) as fourth diagnosis include several combinations of diagnoses in the second and third positions of the trajectories. The subsequent development of chronic ischaemic heart disease (I25) significantly increases the risk that an angina patient will suffer a cardiac arrest (RR=1.13, 95% confidence interval (95% CI): 1.12–1.42, normal distribution approximation *P*=1.38 × 10^−10^). Adding the diagnosis of gout (M10) to the angina and ischaemic heart disease trajectory further increases the RR of cardiac arrest to 1.99 (95% CI: 1.3–3.05, normal distribution approximation *P*=7.66 × 10^−4^). In contrast, and despite numerous studies that demonstrate the impact of ischaemic heart disease on increasing the mortality of patients with renal failure (N18), adding renal failure as a subsequent diagnosis to angina in the trajectory of angina–ischaemic heart disease–cardiac arrest does not further increase the risk for cardiac arrest in the angina patient who develops chronic ischaemic heart disease. Thus, studies designed to assess the effectiveness of strategies to prevent cardiac arrest in angina patients could be tailored to focus on angina patients with both ischaemic heart disease and gout, as these two diagnoses added subsequently to the angina diagnosis markedly increase the likelihood of the outcome of interest (cardiac arrest). See ‘Verifying central diagnoses’ in Methods for the method description and Supplementary Table 1 for the statistic summary.

In a similar manner, the trajectory networks presented offer insights into the complex relationships of end organ pathology of diabetes (E10) in increasing the likelihood of septicaemia (A41), a major complication (Supplementary Table 2). The trajectories demonstrate that the RR of septicaemia is significantly increased by diabetes with subsequent renal failure (N18, RR=3.23, 95% CI: 2.95–3.55, normal distribution approximation *P*=4.99 × 10^−139^) but not by peripheral vascular diseases or vascular complications of the eye (disorders of vitreous body, H43, RR=0.69, normal distribution approximation 95% CI: 0.45–1.04) and retinopathy. However, the co-occurrence of renal failure and disorders of vitreous body in any order subsequent to diabetes results in an RR of septicaemia than renal failure alone of 3.45 (95% CI: 2.08–5.72, normal distribution approximation *P*=7.99 × 10^−7^), which is greater than renal failure alone.

## Discussion

Systematically adding the temporal dimension to population-wide co-morbidity data using a discovery approach on this scale has not been attempted previously. We show for the first time that hospitalizations across an entire population of significant size can be used to extract and group trajectories as a novel way of describing biological disease progression and subsequently identifying keystone diagnoses.

In this work we defined a diagnosis trajectory as an ordered series of diagnoses where the diagnoses were observed in the patients in a specific order. The order had to be observed strictly for a patient to be considered following it. Thus, for a trajectory starting with the diagnoses D1→D2 patients who had the diagnoses assigned in the order D2→D1→D2 again were not considered as following the trajectory. In cases where multiple diagnoses from the trajectory were assigned in the same discharge, they were considered to be in the correct order.

This strict definition puts limits on how much variability each single trajectory is able to cover. It means that bifurcating diagnoses that later converge on other diagnoses are not included in a single trajectory. Nonetheless, using our trajectory clustering approach we were able to cover this type of disease progression as well. Interestingly, using the clustering approach we were further able to reduce the patterns of disease progression from 1,171 individual trajectories down to less than ten major clusters covering most of the populated paths through the disease terminology space. As the underlying data foundation here is considerable and unbiased, this condensation is remarkable. However, it does also obviously reflect the numerical constraints set of the requirement for directionality in terms of RR.

In terms of trajectory interpretation, it is essential eventually to establish to what extent the directionality reflect underlying causal patterns or not. For example, it is interesting to speculate whether the disease state associated with the COPD diagnosis is the cause, or whether COPD is an ICD-10 surrogate for a variety of factors associated with increased morbidity, such as smoking, adverse effects of medications, or poor general health. The expected high degree of association between COPD and atherosclerosis supports the obvious smoking linkage. Hospitalization for pneumonia in the setting of underlying chronic disease is common, with odds ratios reported at 4.4 for COPD and 3.2 for heart failure in one large study^21^. Our model suggests an even more profound effect of these chronic conditions on pneumonia within a relatively short time span. COPD as a subsequent diagnosis of many trajectories may also demonstrate a medical systems issue, namely that of undiagnosed and therefore untreated or undertreated COPD, which becomes manifest only after another serious diagnosis is made. It is likely to be that COPD is coexistent with the initial diagnosis of most trajectories, yet it occurs as a second data point in multiple trajectories. The ability to make data-supported inferences of disease severity and of medical systems issues demonstrates the power of the trajectory analysis. A recent study of 5,812 Danish COPD patients, using extensive questionnaire data, physical examination, lung function and medication data has now provided epidemiologic corroboration, demonstrated a strong, consistent pattern of undertreatment and suggested underdiagnosis of Danes with COPD^22^. The disease trajectories and networks presented here provide the opportunity to conduct a systemic analysis to identify such gaps in disease recognition, diagnosis and treatment.

Our findings demonstrate that the population-wide disease trajectory approach uncovers diagnosis linkages that have had unclear or conflicting relationships through epidemiologic or smaller sample cohort approaches. We further demonstrate the importance of patient stratification and that stratification by type of hospital encounter is as important as stratification by age and gender.

The trajectories have also a predictive potential where preceding steps can be used as a basis for predicting the most probable next step in disease progression. A major additional perspective in using the catalogue of disease trajectories established here is obviously to use them in the context of stratification for precision medicine and combine them with detailed molecular level characterization of each patient, for example, whole-exome or genome sequencing, for better disease management of individual patients along the course each patient will take.

## Methods

### Study design

The objective of this retrospective cohort study was to identify and characterize disease trajectories using population-wide disease registry data using a data-driven approach. The trajectories were derived using pairs of significant time-dependent diagnosis correlations.

The data used in the analysis is from the Danish NPR, which contains administrative information and primary and secondary diagnoses coded in the ICD-10, covering every hospital contact in Denmark. It includes public and private hospital visits and covers all types of encounters: inpatient (admitted to the hospital with overnight stay), outpatient (visit without overnight stay) and emergency department contacts.

The data set covers the period January 1996 to November 2010, and includes 68 million records for 6.2 million individuals. For inpatients, the records cover the time between admission to a ward until discharge to either another ward or out of the hospital. Records covering two or more discharges between wards were combined into one covering the entire admission. In cases of re-admissions the same or the following day after a discharge, the records were also combined. Doing this, 1.5 million inpatient records were combined with other records giving 66.5 million encounters in total. As private hospitals have only reported contacts from 2002, all of these, in total 1 million, were removed to maintain an unbiased data set. Private hospitals in Denmark approximately handle <1.6% of all admissions (routine treatment) and add <1% unique patient diagnosis associations as 38.4% of the patient diagnosis associations from private hospital are already covered by the public hospitals.

The ICD-10 system has a hierarchical structure, where codes can be rounded to a less specific parent diagnosis code, block or chapter. We used this structure to round all codes to level 3 codes.

### Diagnosis correlation measure

We used RR to measure the strength of the correlation between a pair of diagnoses (D1→D2) within 5 years. RR estimates and associated *P*-values were calculated using a sampling approach, where a number of comparison groups were matched to the exposed patients. For this, the exposed group was formed by identifying all discharges with D1 assigned. Comparison groups were formed by matching each of these discharges with a random discharge from the full population. To account for confounding factors, comparison patients were matched to come from the same age and gender group as the exposed patients (as shown in Supplementary Fig. 1). The type of encounter was matched for the D1 discharge. The season of the year and possible changes in diagnostic methods and focus over years were controlled for by sampling the comparison discharge from the same week as the case D1 discharge.

**Figure 5:**
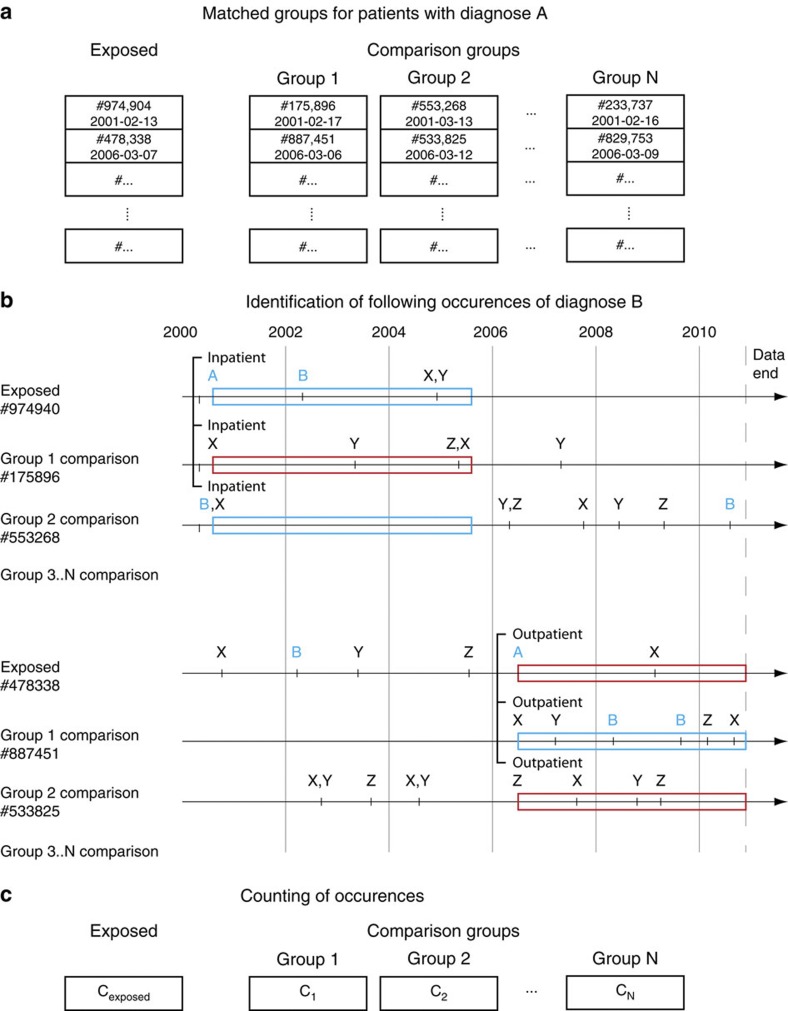
Illustration of the random sampling procedure with *N* samplings for the co-morbidity of diagnosis A followed by diagnosis B within 1 year. (**a**) All discharges with diagnosis A assigned are identified for all patients to make the exposed discharges group. Each exposed discharge is matched with a set of *N* randomly chosen comparison patients with the same gender and age group as the exposed patient and a discharge of the same type in the same week. Each line in **a** shows a single exposed patient discharge and its matched comparison patient discharge. (**b**) The diagnosis history of the exposed and comparison cases and controls is examined to see whether diagnosis B occurred within 1 year of the matched week in which the case had diagnosis A (a blue box indicates that diagnosis B occurs within the time frame). X, Y and Z represents arbitrary other diagnoses. (**c**) The number of these occurrences is counted for each cohort giving a number of overlaps. The count for the cases is the observed overlap, while the control cohorts are used to estimate the *P*-values.

We sampled a number (*N*) of comparison groups. Subsequently, D1 discharges that have one or more subsequent D2 discharges within a 5-year time frame were counted for all groups (Fig. 5). We denote the number for the exposed group as *C*_exposed_ and as *C*_*i*_, where *i*=1. *N* is for the comparison groups. RR is given by,


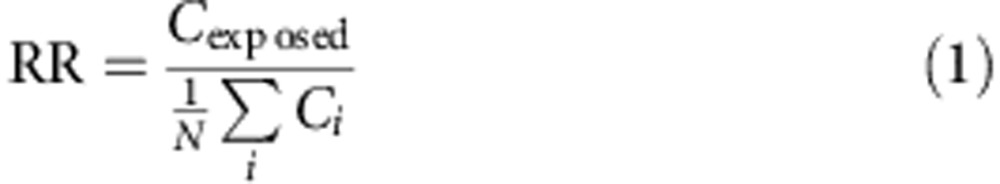


The *P*-value was calculated as the percentage of the comparison groups with a total co-occurrence counts that is larger than the observed co-occurrence count,


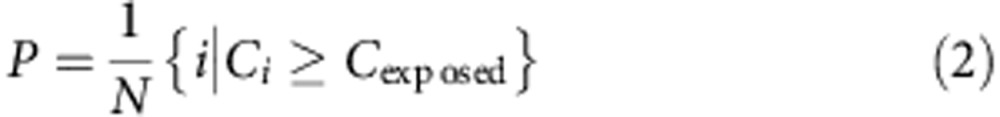


RR was estimated using 10,000 sampled comparison groups for each pair of diagnoses. Owing to correction for multiple testing for 823,606 pairs, we needed more than 82 million samples for each pair to obtain a significant *P*-value. A binomial model was used as a pre-filtering step to avoid a total running time of several thousands of computing years if performing the full procedure. Pairs included in the trajectories were validated using the full sampling procedure.

In the binomial test modelling the sampling procedure, we considered each sampling of a single comparison discharge as a Bernoulli trial. Given the matching criteria, there is a set of *n*_match_ discharges to select the random comparison group discharge from. A number, *c*, of these discharges will have D2 discharge within the time frame. This number can be pre-calculated without any sampling. The probability of sampling a comparison group discharge with a D2 discharge within the time frame is,


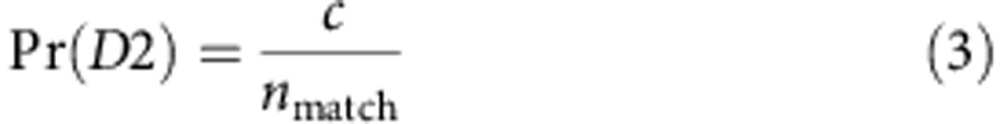


The probability distribution for the total number of sampled D2 discharges is the sum of all single Bernoulli trials. We approximated the distribution with a single binomial test that uses the average of the probabilities for all D1 discharges as probability parameter,


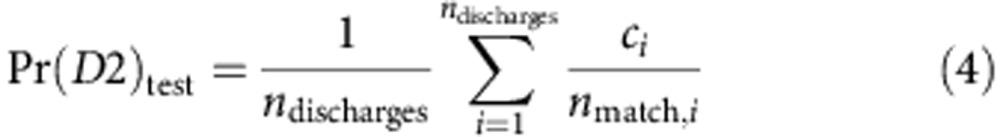


where *n*_discharges_ is the number of discharges with D1.

**Figure 6:**
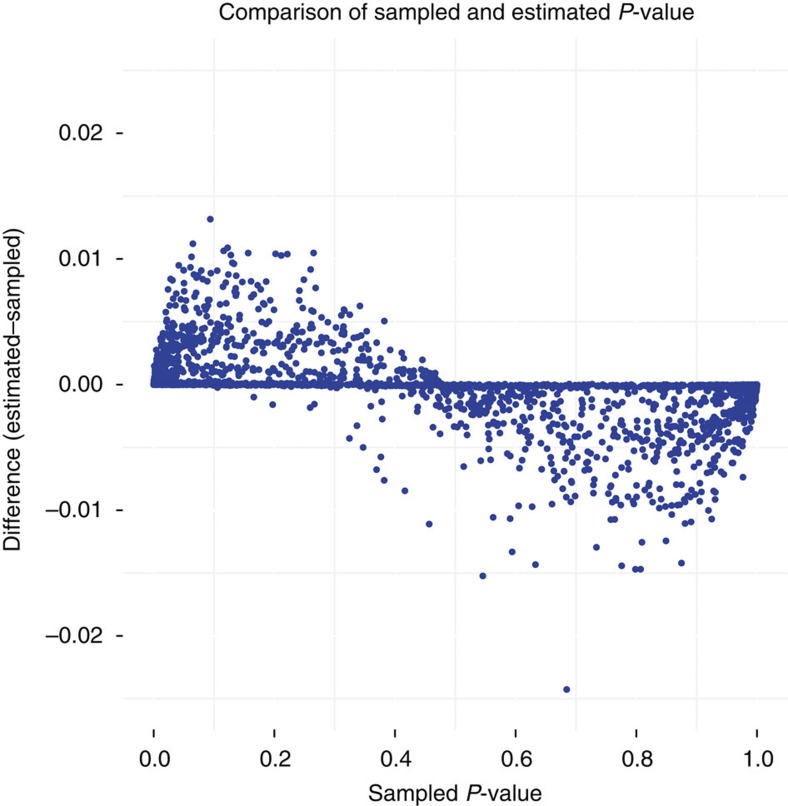
Validation of *P*-values estimated with binomial testing. Each point represents the sampled *P*-value and the difference between estimated and sampled for a pair of diagnoses for some time limit. The estimated *P*-values are from the model with one population average and positive difference implies that the estimated model is more conservative. The fact that the estimated model is more conservative for small *P*-values reduced the likelihood that using the estimate will cause a false positive. As an extra precaution against false positives, we used a *P*-value cut-off of 0.001 (before correction) when using the binomial estimated *P*-values.

To make sure the binomial model is a valid substitution for the sampling giving *P*-values that are at least as conservative as the sampling procedure, we ran full sampling for 1,500 pairs and compared with the simplification. We expect the simplification to perform worst where the variance of the probabilities contributing to the average probability is high. Therefore, we tested the 1,000 pairs with the largest variance, while 500 others were chosen at random. Figure 6 shows the true *P*-values plotted against estimated *P*-values. The binomial model was found to be more conservative than the sampled *P*-values for small *P*-value. Thus, the simplification is a valid substitute for the sampling procedure. To further guard against false positives due to the binomial model, the significance cutoff level was set to 0.001.

### Testing for directionality

The diagnosis pairs (D1, D2) that had RR>1 and a significant *P*-value for one or both directions (D1→D2 and/or D2→D1) were tested for directionality. Binomial tests were used to identify pairs where significantly more patient had D1 assigned before D2 or the other way around. For this, the first D1 and D2 discharges for patients with both diagnoses were identified and the order for each patient established. The number of patients with each order of the diagnoses was counted: *N*_D1_ with D1 assigned first, *N*_D2_ with D2 assigned first and *N*_same_ with D1 and D2 in the same discharge. Using two binomial tests, we tested whether *N*_D1_ or *N*_D2_ were significantly larger compared with a binomial distribution (*N*_D1_+*N*_same_+*N*_D2_ samples with probability 50%). The *P*-values were Bonferroni corrected. If one of the tests showed a *P*-value <0.05, the pair was considered having a significant direction (only one of the tests can have significant *P*-value).

### Diagnosis trajectories

We counted the patients as following a disease trajectory only if the patient had the diagnoses assigned strictly in the order specified by the trajectory. Each step of the trajectory corresponded to a single diagnosis.

We used the pairs of diagnoses from the temporal correlation analysis with significant direction to identify the trajectories to count. Trajectories with three diagnoses were obtained by combining pairs with overlapping diagnoses (D1→D2 and D2→D3 combined to D1→D2→D3). They were subsequently extended with more overlapping pairs to obtain even longer trajectories. A greedy approach was used to find the three long trajectories covering the most patients. The pairs were sorted in descending order according to their discharge count. Pairs with an overlapping diagnosis were found starting from the top of the list and the number of patients following the full trajectory was counted. We stopped when the three long trajectories had no patients following them. All four and five long trajectories were then counted. Using this approach, all the trajectories covering the largest number of patients can be identified without counting every possible trajectory.

To establish the robustness of the trajectories, we calculated a bootstrap value for each trajectory by resampling the population. As the cutoff for a trajectory to be in the final set was a minimum of 20 patients, the bootstrap value is the proportion of the trajectories that have 20 or more patients in the resampled populations. In our case, this bootstrap approach is equivalent to a series of Bernoulli trials where a patient is drawn from the population and success is defined as drawing a patient who follows the trajectory. Given a trajectory with *N* patients in the last step and a population of *N*_pop_=6.2 million, the randomly sampled trajectory count (*C*) follows a binomial distribution with *C*~B(*n*_trials_=*N*_pop_, *P*=*N/N*_pop_). The bootstrap support will thus converge to the probability P(*C*≥20) as the number of bootstrapped samples approach infinity. Consequently, the support can be calculated through a binomial test and will be identical for all trajectories that are followed by the same number of patients.

### Comparison with co-morbidities from Stockholm, Sweden

To validate that the diagnosis pairs within the trajectories are not unique to the Danish population, we compared them against a study of co-morbidities in a cohort of almost 600,000 individuals from the greater Stockholm area^11^. We used the top 1000 co-morbidities, which are made available through Comorbidity-View (http://www2.dsv.su.se/comorbidityview-demo/, accessed: 25 February 2014). Our trajectories consist of 140 unique diagnoses, which can be combined into 9,730 unique undirected pairs. The trajectories contain 1,155 unique pairs, if for a trajectory D1→…→D4 we include all six possible combinations of pairs ([D1,D2], [D1,D3], [D1,D4], [D2,D3], [D2,D4], [D3,D4]). Of the top 1,000 pairs in the Swedish study, 221 pairs consist of 2 diagnoses that can be found in our trajectories, 106 of which (48%) fall within the set of 1,155 trajectory pairs. We compared this with random expectation using a binomial test B(*n*_trials_=206, *P*=9,730/1,115).

### Diagnosis trajectory clustering

In the 1,171 four long diagnosis trajectories we identified groups of trajectories having large diagnosis overlap and representing variants of general patterns of disease progression. To identify these patterns systematically, we used the Markov Cluster Algorithm^23^ that assigned each of the 140 codes that make up the 1,171 trajectories to a cluster. The Jaccard index was used as similarity measure (counting how many trajectories both diagnoses are part of and normalizing by the total number of trajectories either is part of). Trajectories with all diagnoses within the same cluster were combined into directed trajectory clusters in which the patterns could be examined (Figs 2, 3, 4).

As the clustering was based on diagnoses, some trajectories had diagnoses from multiple clusters. Of the original 1,171 trajectories, there were 378 that had all diagnoses within the same cluster. We increased this number to 608 by merging one smaller cluster into the largest and by including particular diagnoses to clusters if they contributed to complete trajectories with three diagnoses already within the cluster. In this way, some diagnoses appear in multiple clusters. Of the 608 trajectories, 466 were within the largest cluster, 129 within the second largest cluster, 6 within the third largest, 5 within the fourth largest and 2 in each their cluster. The second largest through the fourth largest cluster each revealed a distinct pattern of disease progression (the COPD, cerebrovascular and prostate cancer patterns), whereas the largest cluster had two major patterns in it: one focusing on diabetes mellitus and another focusing on cardiovascular diagnoses.

To divide the largest cluster into the two patterns, the diagnoses within it were once again clustered using MCL. We used the same similarity measure as before, but using larger inflation factor. This resulted in four new sub-clusters, where the largest subcluster covered diabetes mellitus diagnoses. We merged the second and third largest subclusters, which together covered cardiovascular diagnoses.

Finally, the five clusters were visualized by representing diagnoses as nodes and making directed edges between consecutive diagnoses for all the trajectories within the same cluster.

### Verifying central diagnoses

In most of the trajectory clusters, we identified a key diagnosis. To verify that they are central to the disease progression in the clusters, we, for each key diagnosis, counted how often it occurred between diagnoses preceding it and diagnoses succeeding it in the full population. We identified two sets of diagnoses: all diagnoses that could lead to the key diagnoses (the preceding set) and all diagnoses that could be reached from the key diagnoses (the succeeding set). Next, we identified all exposed patients who had one diagnosis from the preceding set followed by one from the succeeding set. Similar to when counting the trajectories, we discarded patients who have a diagnosis from the succeeding set before the first from the preceding set, and the diagnoses were allowed to occur in the same admission. We then counted the patients having their first occurrences of the key diagnosis in the time from the first occurrence of a preceding diagnosis to the first occurrence of a succeeding diagnosis.

To evaluate the count of the key diagnosis, we calculated RR and assigned *P*-values by matching comparison groups using the same criteria as for the temporal correlation analysis. For each exposed patient, we identified the number of days between the occurrences of the preceding diagnosis to the occurrence of the succeeding diagnosis. We counted the number of occurrences of the key diagnosis in the same period among the matched comparison patients. The findings are summarized in Supplementary Table 3.

In addition, we investigated which combination of key diagnoses could lead to severe outcome in the cardiovascular and diabetes trajectory groups. Severe outcome was defined as cardiac arrest in the cardiovascular network and as septicaemia in the diabetes trajectory group. Patients following a trajectory starting with angina pectoris (cardiovascular trajectory group) or insulin-dependent diabetes mellitus were stratified on the presence of all possible combinations of key diagnoses within trajectories leading to this outcome. For each combination of diagnoses, we counted the number of patients with and without the severe outcome, and RR was calculated. *P*-values were approximated with a normal distribution. Supplementary Tables 1 and 2 shows the statistics for the cardiovascular trajectory group and the diabetes trajectory group, respectively.

### Data and materials approval

The NPR registry data is protected by the Danish Act on Processing of Personal Data and can only be accessed following application. This study has been approved by Danish Data Registration Agency, Copenhagen (ref: 2010–54–1059) and the National Board of Health, Copenhagen (ref: 7–505–29–1624/1).

## Author contributions

Planning and design was done by A.B.J., S.B. and L.L.J. P.B.J. assisted with the initial ideas and input throughout the process. All computational analyses were done by A.B.J. The manuscript was written mainly by A.B.J., L.L.J., P.L.M. and S.B. and was read and commented by all co-authors. Analysis and condensation of results was done mainly by P.L.M., T.I.O., L.L.J. and A.B.J. S.G.E., R.E. and H.S. have contributed in the early stages of analysis.

## Additional information

**How to cite this article:** Jensen, A. B. *et al*. Temporal disease trajectories condensed from population-wide registry data covering 6.2 million patients. *Nat. Commun.* 5:4022 doi: 10.1038/ncomms5022 (2014).

## Supplementary Material

Supplementary Figure and Tables Supplementary Figure 1 and Supplementary Tables 1-3

Supplementary Data 1 Directional diagnosis pairs. All 4,014 pairs from the temporal analysis that were significant (sampling method *p* < 2.21*10^-9^) with RR > 1 and had significant directionality (binomial test *p* < 6.037*10^-7^) are given in the table. Diagnosis codes for the pair (D1 → D2) and statistics are given: Number of patients D1 assigned before D2, RR, associated p-value for the estimated binomial model and the sampled p-value with the number of samples used to obtain it, and the p-value for directionality.

Supplementary Data 2 Trajectory counting. The table reports all 1,171 length 4 trajectories along with patient counts, bootstrap support, median age at onset of first diagnosis and median duration from onset of first diagnosis to each subsequent step.

## Figures and Tables

**Table 1:** Number of significant pairs (binomial test *P*<1.21 × 10^−9^) in the temporal analysis given different combinations of patient stratification.

	**Age and gender**	**Not age and gender**
Type of encounter	62,821	72,832
Not type of encounter	72,937	81,058

Stratifying for either type of hospital encounter or age and gender reduces the total number of significant pairs by 8,121 and 8,226, respectively, compared with no stratification, and by 18,237 when stratifying for both. This indicates that stratification for type of hospital encounter is as important as stratification by age and gender, and that the two contribute independently.

## References

[b1] Camilo O. & Goldstein L. B. Seizures and epilepsy after ischemic stroke. Stroke 35, 1769–1775 (2004). 15166395 10.1161/01.STR.0000130989.17100.96

[b2] Finkelstein J., Cha E. & Scharf S. M. Chronic obstructive pulmonary disease as an independent risk factor for cardiovascular morbidity. Int. J. COPD 4, 337–349 (2009). 10.2147/copd.s6400 PMC2754086 19802349

[b3] Teno J. M., Weitzen S., Fenell M. L. & Mor V. Dying trajectory in the last year of life: does cancer trajectory fit other diseases? J. Palliat. Med. 4, 457–464 (2001). 11798477 10.1089/109662101753381593

[b4] Murtagh F. E. M., Murphy E. & Sheerin N. S. Illness trajectories: an important concept in the management of kidney failure. Nephrol. Dialysis Transplant 23, 3746–3748 (2008). 10.1093/ndt/gfn532 18809974

[b5] Murtagh F. E. M., Sheerin N. S., Addington-Hall J. & Higginson I. J. Trajectories of illness in stage 5 chronic kidney disease: a longitudinal study of patient symptoms and concerns in the last year of life. Clin. J. Am. Soc. Nephrol. 6, 1580–1590 (2011). 21685021 10.2215/CJN.09021010

[b6] Murray S. A., Kendall M., Boyd K. & Sheikh A. Illness trajectories and palliative care. BMJ 330, 1007–1011 (2005). 15860828 10.1136/bmj.330.7498.1007 PMC557152

[b7] Petri H., Maldonato D. & Robinson N. J. Data-driven identification of co-morbidities associated with rheumatoid arthritis in a large US health plan claims database. BMC Musculoskelet. Disord. 11, 247 (2010). 20973999 10.1186/1471-2474-11-247 PMC2987972

[b8] Blair D. R. . A nondegenerate code of deleterious variants in Mendelian loci contributes to complex disease risk. Cell 155, 70–80 (2013). 24074861 10.1016/j.cell.2013.08.030 PMC3844554

[b9] Hidalgo C. A., Blumm N., Barabási A.-L. & Christakis N. A. A dynamic network approach for the study of human phenotypes. PLoS Comput. Biol. 5, e1000353 (2009). 19360091 10.1371/journal.pcbi.1000353 PMC2661364

[b10] Chen L. L., Blumm N., Christakis N. A., Barabási A.-L. & Deisboeck T. S. Cancer metastasis networks and the prediction of progression patterns. Br. J. Cancer 101, 749–758 (2009). 19707203 10.1038/sj.bjc.6605214 PMC2736851

[b11] Tanushi H., Dalianis H. & Nilsson G. H. Calculating Prevalence of Comorbidity and Comorbidity Combinations with Diabetes in Hospital Care in Sweden Using a Health Care Record Database. In:Proceedings of LOUHI 2011 Third International Workshop on Health Document Text Mining and Information Analysis 59–65Bled, Slovenia (2011).

[b12] Curkendall S. M. . Cardiovascular disease in patients with chronic obstructive pulmonary disease, Saskatchewan Canada cardiovascular disease in COPD patients. Ann. Epidemiol. 16, 63–70 (2006). 16039877 10.1016/j.annepidem.2005.04.008

[b13] Sidney S. . COPD and incident cardiovascular disease hospitalizations and mortality: Kaiser Permanente Medical Care Program. Chest 128, 2068–2075 (2005). 16236856 10.1378/chest.128.4.2068

[b14] Salisbury A. C., Reid K. J. & Spertus J. A. Impact of chronic obstructive pulmonary disease on post-myocardial infarction outcomes. Am. J. Cardiol. 99, 636–641 (2007). 17317363 10.1016/j.amjcard.2006.09.112

[b15] Suissa S., Dell’Aniello S. & Ernst P. Long-term natural history of chronic obstructive pulmonary disease: severe exacerbations and mortality. Thorax 67, 957–963 (2012). 22684094 10.1136/thoraxjnl-2011-201518 PMC3505864

[b16] Moss S. E., Klein R., Klein B. E. K. & Wong T. Y. Retinal vascular changes and 20-year incidence of lower extremity amputations in a cohort with diabetes. Arch. Intern. Med. 163, 2505–2510 (2003). 14609788 10.1001/archinte.163.20.2505

[b17] Kohner E. M. Diabetic retinopathy. Br. Med. Bull. 45, 148–173 (1989). 2477110 10.1093/oxfordjournals.bmb.a072309

[b18] Freedman D. S., Williamson D. F., Gunter E. W. & Byers T. Relation of serum uric acid to mortality and ischemic heart disease: the NHANES I Epidemiologic Follow-up Study. Am. J. Epidemiol. 141, 637–644 (1995). 7702038 10.1093/oxfordjournals.aje.a117479

[b19] Kelkar A., Kuo A. & Frishman W. H. Allopurinol as a cardiovascular drug. Cardiol. Rev. 19, 265–271 (2011). 21983313 10.1097/CRD.0b013e318229a908

[b20] Yang Q. . Multiple genetic loci influence serum urate and their relationship with gout and cardiovascular disease risk factors. Circ. Cardiovasc. Genet. 3, 523–530 (2010). 20884846 10.1161/CIRCGENETICS.109.934455 PMC3371395

[b21] Farr B. M., Bartlett C. L., Wadsworth J. & Miller D. L. Risk factors for community-acquired pneumonia diagnosed upon hospital admission. British Thoracic Society Pneumonia Study Group. Respir. Med. 94, 954–963 (2000). 11059948 10.1053/rmed.2000.0865

[b22] Ingebrigtsen T. S. . Characteristics of undertreatment in COPD in the general population. Chest 144, 1811–1818 (2013). 23989916 10.1378/chest.13-0453

[b23] van Dongen S. Graph clustering by flow simulation PhD thesis (Univ. Utrecht (2000).

