# Supplementary material for: Temporal disease trajectories condensed from population-wide registry data covering 6.2 million patients
Source: Nat Commun. 2014 Jun 24;5:4022. doi: 10.1038/ncomms5022 (PMC4090719; doi:10.1038/ncomms5022)
Supplement: Supplementary Figure and Tables — Supplementary Figure 1 and Supplementary Tables 1-3 [file ncomms5022-s1.pdf]

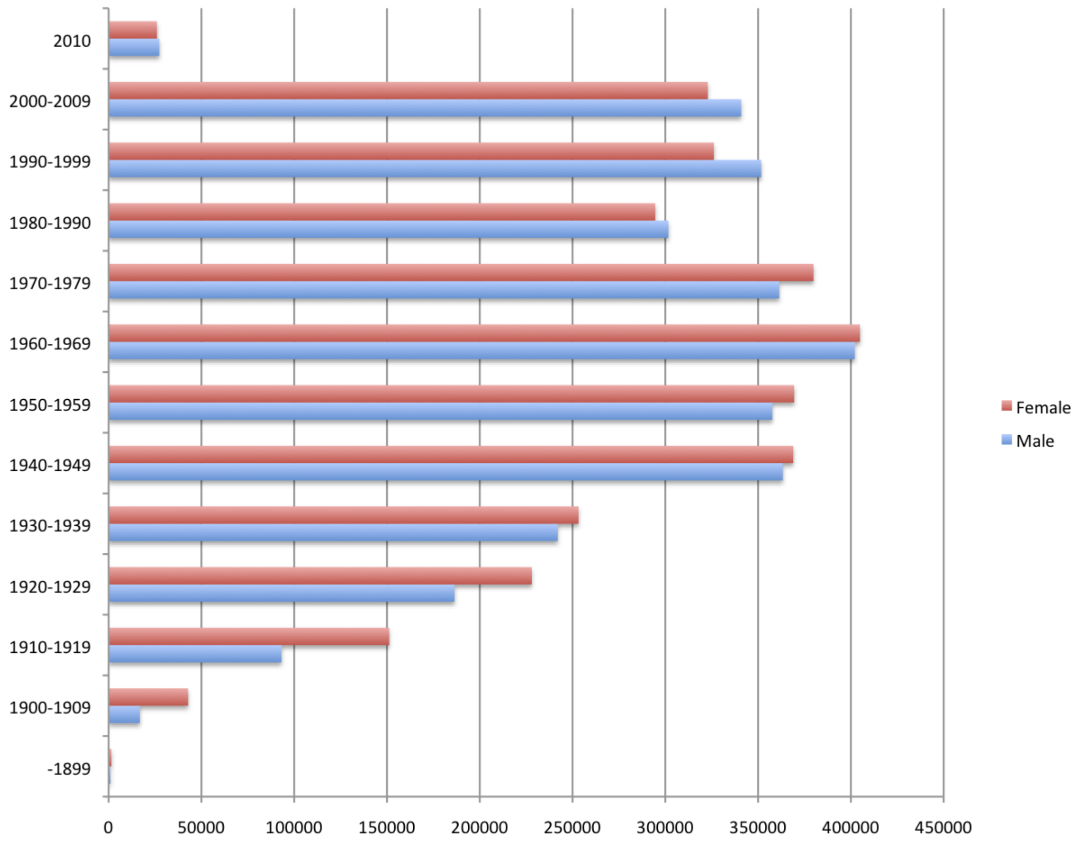

S**upplementary Figure 1 |** The NPR population distributed over gender and age (birth decade). The bins shown were used for stratification when matching patients for the comparison groups. For the temporal correlation analysis patients born before 1900 and in 2010 are removed from analyzes using the binning, as the subpopulations are too small to give adequate statistical results. However, the full population is used when counting the trajectories.

Key diagnoses	Ends with CA Yes	No	RR	RR 95% CI Low	High	P-value
No key-diagnose	1423	121603	-	-	-	
I25	1015	65860	1.31	1.21	1.42	1.382E-11
I50+I25	582	16582	2.93	2.67	3.22	3.741E-109
I50	334	10891	2.57	2.29	2.89	3.497E-56
I50+I25+J18	230	6292	3.05	2.66	3.50	1.593E-57
J18	155	9133	1.44	1.22	1.70	6.230E-06
I25+J18	141	8094	1.48	1.25	1.76	3.731E-06
I50+J18	98	3064	2.68	2.19	3.28	4.792E-22
K29	36	3747	0.82	0.59	1.14	8.773E-01
I50+K29+I25	35	1153	2.55	1.83	3.54	1.465E-08
M10+I50+I25	34	709	3.96	2.84	5.52	2.540E-16
K29+I25	32	3086	0.89	0.63	1.26	7.494E-01
M10+I50+I25+J18	26	486	4.39	3.01	6.41	8.598E-15
I50+K29+I25+J18	21	693	2.54	1.66	3.89	8.206E-06
M10+I25	21	891	1.99	1.30	3.05	7.660E-04
K29+I25+J18	16	670	2.02	1.24	3.28	2.381E-03
I50+K29	11	513	1.81	1.01	3.26	2.329E-02
M10	10	961	0.89	0.48	1.65	6.435E-01
K29+J18	9	544	1.41	0.73	2.70	1.516E-01
M10+I50	7	303	1.95	0.94	4.07	3.707E-02
M10+I50+K29+I25	4	83	3.97	1.52	10.37	2.389E-03
M10+I50+J18	4	133	2.52	0.96	6.64	3.027E-02
M10+J18	4	215	1.58	0.60	4.18	1.786E-01
I50+K29+J18	4	235	1.45	0.55	3.83	2.284E-01
M10+I25+J18	4	288	1.18	0.45	3.14	3.669E-01
M10+K29+I25+J18	2	27	5.96	1.56	22.73	4.462E-03
M10+I50+K29	2	30	5.40	1.41	20.70	6.903E-03
M10+I50+K29+J18	1	26	3.20	0.47	21.93	1.179E-01
M10+K29+J18	1	27	3.09	0.45	21.17	1.255E-01
M10+K29	1	59	1.44	0.21	10.07	3.563E-01
M10+K29+I25	0	69	NA	NA	NA	NA
M10+I50+K29+I25+J18	0	84	NA	NA	NA	NA

ICD-10 code	Diagnosis name
I25	Chronic ischaemic heart disease
I50	Heart failure
J18	Pneumonia, organism unspecified
K29	Gastritis and duodenitis
M10	Gout

Supplementary Table 1 | Statistics for diagnoses leading to cardiac arrest (CA). The counting covers 260,841 patients who follow a trajectory starting with angina pectoris. Patients are stratified by which combination of key diagnoses followed the angina pectoris diagnosis. The number of patient with a trajectory ending with CA is reported for each combination. The RR of having the combination compared to having no diagnosis from the set of all key diagnoses is given for each combination with a 95% confidence interval and a p-value (normal distribution approximation).

Supplementary Table 2

	Ends with septicemia			RR 95% CI		
**Key diagnoses**	**Yes**	**No**	**RR**	**Low**	**High**	**P-value**
No key-diagnose	3810	60837	-	-	-	-
N18	416	1766	3.23	2.95	3.55	4.992E-139
H43	21	499	0.69	0.45	1.04	3.891E-02
N18+H43	12	47	3.45	2.08	5.72	7.986E-07
D64	355	2856	1.88	1.69	2.08	1.982E-33
N18+D64	83	442	2.68	2.20	3.28	1.827E-22
H43+D64	5	37	2.02	0.89	4.60	4.708E-02
N18+H43+D64	2	8	3.39	0.98	11.73	2.672E-02
E16	353	7174	0.80	0.72	0.89	1.285E-05
N18+E16	77	296	3.50	2.86	4.28	1.507E-34
H43+E16	10	93	1.65	0.91	2.97	4.856E-02
N18+H43+E16	2	24	1.31	0.34	4.94	3.475E-01
D64+E16	52	524	1.53	1.18	1.99	6.833E-04
N18+D64+E16	23	109	2.96	2.04	4.29	5.942E-09
H43+D64+E16	1	12	1.31	0.20	8.58	3.908E-01
N18+H43+D64+E16	1	7	2.12	0.34	13.27	2.108E-01
H36	277	8608	0.53	0.47	0.60	1.160E-25
N18+H36	67	373	2.58	2.07	3.23	3.200E-17
H43+H36	46	794	0.93	0.70	1.23	3.053E-01
N18+H43+H36	31	115	3.60	2.63	4.93	6.127E-16
D64+H36	50	451	1.69	1.30	2.21	4.831E-05
N18+D64+H36	27	126	2.99	2.12	4.22	1.996E-10
H43+D64+H36	5	58	1.35	0.58	3.12	2.441E-01
N18+H43+D64+H36	7	32	3.05	1.56	5.96	5.783E-04
E16+H36	83	2204	0.62	0.50	0.76	4.245E-06
N18+E16+H36	30	174	2.50	1.79	3.48	3.341E-08
H43+E16+H36	16	274	0.94	0.58	1.51	3.932E-01
N18+H43+E16+H36	27	65	4.98	3.62	6.85	2.603E-23
D64+E16+H36	17	172	1.53	0.97	2.40	3.415E-02
N18+D64+E16+H36	9	70	1.93	1.04	3.58	1.796E-02
H43+D64+E16+H36	6	36	2.42	1.15	5.09	9.628E-03
N18+H43+D64+E16+H36	6	22	3.64	1.79	7.40	1.827E-04
I73	104	1783	0.94	0.77	1.13	2.439E-01
N18+I73	35	156	3.11	2.30	4.20	7.513E-14
H43+I73	2	37	0.87	0.23	3.36	4.200E-01
N18+H43+I73	5	7	7.07	3.62	13.82	5.324E-09
D64+I73	21	162	1.95	1.30	2.92	6.061E-04
N18+D64+I73	9	62	2.15	1.17	3.96	7.033E-03
H43+D64+I73	1	7	2.12	0.34	13.27	2.108E-01

	Ends with septicemia			RR 95% CI		
Key diagnoses	Yes	No	RR	Low	High	P-value
No key-diagnose	3810	60837	-	-	-	-
N18+H43+D64+I73	1	5	2.83	0.47	16.93	1.274E-01
E16+I73	18	336	0.86	0.55	1.35	2.607E-01
N18+E16+I73	11	44	3.39	2.00	5.76	3.046E-06
H43+E16+I73	0	4	NA	NA	NA	NA
N18+H43+E16+I73	1	0	16.97	16.45	17.50	0.000E+00
D64+E16+I73	3	39	1.21	0.41	3.61	3.649E-01
N18+D64+E16+I73	5	15	4.24	1.98	9.07	9.652E-05
H43+D64+E16+I73	0	0	NA	NA	NA	NA
N18+H43+D64+E16+I73	1	1	8.48	2.12	33.93	1.251E-03
H36+I73	28	432	1.03	0.72	1.48	4.303E-01
N18+H36+I73	9	61	2.18	1.18	4.02	6.146E-03
H43+H36+I73	4	63	1.01	0.39	2.62	4.894E-01
N18+H43+H36+I73	5	18	3.69	1.70	8.01	4.893E-04
D64+H36+I73	6	52	1.76	0.82	3.75	7.293E-02
N18+D64+H36+I73	4	23	2.51	1.02	6.21	2.295E-02
H43+D64+H36+I73	1	7	2.12	0.34	13.27	2.108E-01
N18+H43+D64+H36+I73	1	7	2.12	0.34	13.27	2.108E-01
E16+H36+I73	5	136	0.60	0.25	1.42	1.239E-01
N18+E16+H36+I73	6	34	2.55	1.22	5.33	6.572E-03
H43+E16+H36+I73	0	18	NA	NA	NA	NA
N18+H43+E16+H36+I73	1	9	1.70	0.26	10.90	2.887E-01
D64+E16+H36+I73	2	29	1.09	0.29	4.18	4.474E-01
N18+D64+E16+H36+I73	1	11	1.41	0.22	9.24	3.588E-01
H43+D64+E16+H36+I73	0	7	NA	NA	NA	NA
N18+H43+D64+E16+H36+I73	3	5	6.36	2.60	15.57	2.541E-05

ICD-10 code	Diagnosis name
N18	Chronic renal failure
H43	Disorders of vitreous body
D64	Other anaemias
E16	Other disorders of pancreatic internal secretion
H36	Retinal disorders in diseases classified elsewhere
I73	Other peripheral vascular diseases

Supplementary Table 2 | Statistics for diagnoses leading to septicemia. Similar to Supplementary Table 1, but for 98,095 diabetes patients with septicemia as the end diagnosis.

Key diagnose	Have pattern	Have key diagnosis		Comparison	RR	p-value
G40 Epilepsy	41,681	2,902	(6.96%)	441.9	6.6	$< 10^{-5}$
H36 Retina disorder	66,758	5,255	(7.87%)	261.9	20.1	$< 10^{-5}$
J44 COPD	156,403	14,460	(9.25%)	2813.4	5.1	$< 10^{-5}$
M10 Gout	105,878	2,470	(2.33%)	363.5	6.8	$< 10^{-5}$

Supplementary Table 3 | Statistics on key diagnoses in trajectory clusters. The table shows counts of how many patients had the pattern (preceding diagnosis followed by succeeding diagnosis), how many of those had the key diagnosis in the period between the preceding and succeeding diagnosis, how many occurrences of the key diagnosis were found on average among the matched comparison groups and RR and p-value for this. 10,000 sampled comparison groups were used to assess RR and p-values (sampling method).
